# A phase II trial of ixabepilone in Asian patients with advanced gastric cancer previously treated with fluoropyrimidine-based chemotherapy

**DOI:** 10.1007/s00280-012-1943-6

**Published:** 2012-08-12

**Authors:** Yeul Hong Kim, Kei Muro, Hirofumi Yasui, Jen-Shi Chen, Min-Hee Ryu, Se-Hoon Park, Kent-Man Chu, Su-Pin Choo, Teresa Sanchez, Christine DelaCruz, Pralay Mukhopadhyay, Ioannis Lainas, Chung-Pin Li

**Affiliations:** 1 Division of Oncology and Hematology, Department of Internal Medicine, Korea University College of Medicine, Seoul, Korea; 2 Department of Clinical Oncology, Aichi Cancer Center Hospital, Aichi, Japan; 3 Division of Gastrointestinal Oncology and Endoscopy, Shizuoka Cancer Center, Sunto-gun, Japan; 4 Division of Hematology-Oncology, Department of Internal Medicine, Chang Gung Memorial Hospital and Chang Gung University, Taoyuan County, Taiwan; 5 Department of Oncology, University of Ulsan College of Medicine, Asan Medical Center, Seoul, Korea; 6 Division of Hematology-Oncology, Department of Medicine, Samsung Medical Center, Sungkyunkwan University School of Medicine, Seoul, Korea; 7 Department of Surgery, The University of Hong Kong, Hong Kong, People’s Republic of China; 8 Department of Medical Oncology, National Cancer Centre, Singapore, Singapore; 9 Bristol-Myers Squibb, Princeton, NJ USA; 10 Division of Gastroenterology, Department of Medicine, Taipei Veterans General Hospital, No. 201, Sec. 2, Shih-Pai Road, Taipei, 11217 Taiwan; 11 National Yang-Ming University School of Medicine, Taipei, Taiwan; 12 Bristol-Myers Squibb, Singapore, Singapore; 13 Bristol-Myers Squibb, Braine L’Alleud, Belgium

**Keywords:** Gastric cancer, Second-line therapy, Asian patients, Ixabepilone

## Abstract

**Purpose:**

The highest rates of gastric cancer occur in Eastern Asia. Fluoropyrimidine-based therapy is used initially in unresectable and metastatic disease, but no single standard of care exists following disease progression. Ixabepilone, an epothilone B analog, is a non-taxane microtubule-stabilizing agent with clinical activity across multiple tumor types approved by the United States Food and Drug Administration for treatment of metastatic breast cancer.

**Methods:**

Asian patients with unresectable or metastatic gastric adenocarcinoma who had failed fluoropyrimidine-based chemotherapy received ixabepilone 40 mg/m^2^ by 3-h intravenous infusion every 3 weeks. The primary endpoint was objective response rate (ORR).

**Results:**

Fifty-two patients were treated (65.4 % men; median age: 56.5 years). The ORR was 15.4 % (95 % confidence interval [CI] 6.9–28.1); 8 patients achieved partial responses for a median duration of 3.1 months (95 % CI 2.6–4.1 months) and 26 patients (50.0 %) had stable disease. Median progression-free survival was 2.8 months (95 % CI 2.1–3.5 months). The most common grade 3 non-hematological toxicities were fatigue (9.6 %), decreased appetite (7.7 %), sensory neuropathy (5.8 %), and diarrhea (5.8 %). Grade 3/4 neutropenia occurred in 46.2 % of patients.

**Conclusions:**

Ixabepilone is active in Asian patients with advanced gastric cancer and shows a toxicity profile similar to those previously reported in other tumor types.

## Introduction

Gastric cancer was newly diagnosed in an estimated 989,600 people and caused an estimated 738,000 deaths worldwide in 2008 [1]; it was the third leading cause of cancer deaths in men and fifth leading cause in women. The highest rates of gastric cancer occur in Eastern Asia, where the age-standardized incidence is 42.4 per 100,000 among men and 18.3 per 100,000 among women [1]. Surgery with curative intent is the mainstay of treatment in localized disease, with perioperative chemotherapy or adjuvant chemoradiation or chemotherapy for patients with stage II or III disease depending on national standards [2–4]. However, more than two-thirds of patients have unresectable disease at the time of diagnosis and 60 % of resectable cases eventually relapse [5, 6]. Non-curative gastrectomy may be used in palliation, but it is associated with high rates of procedure-related morbidity and mortality as well as poor 1-year survival [7].

In the metastatic disease setting, combination chemotherapy with regimens containing a fluoropyrimidine and a platinum agent is widely used initially, with a third cytotoxic agent often included for medically fit patients [2, 3]. Nevertheless, even with the most active regimens, progression-free survival (PFS) remains in the range of 5–7 months and median survival is only 9–11 months [8–11]. In Japan, cisplatin plus the oral fluoropyrimidine S–1 has emerged as a preferred first-line regimen producing median survival of 13 months [12]. Following progression, 20–40 % of patients in Western countries subsequently receive second-line chemotherapy [13], but the number is higher (60–70 %) in Asian countries, particularly Japan and Korea. There is no established second-line regimen; options include paclitaxel, docetaxel, or irinotecan given alone or in doublet regimens, which produced median survival of 4–8 months in prospective clinical trials [14–18]. These survival data underscore the need for more effective therapy in metastatic gastric cancer.

Ixabepilone is the first member of the epothilone class of microtubule-stabilizing drugs to be approved for use in cancer therapy, specifically monotherapy or in combination with capecitabine for treatment of recurrent breast cancer [19, 20]. Ixabepilone is structurally distinct from the taxanes because it binds to a different site on β-tubulin and has reduced susceptibility to common mechanisms that confer resistance to taxanes and other anti-cancer drugs [21, 22]. Phase II clinical studies have demonstrated that ixabepilone has activity against a wide range of tumor types besides breast cancer, including hormone-refractory prostate cancer [23, 24], pancreatic cancer [25], non-small cell lung cancer [26], endometrial carcinoma [27], ovarian cancer [28], and renal cell carcinoma [29].

Ixabepilone administered every 3 weeks produced an objective response rate (ORR) of 5 or 9 % in Western patients with metastatic gastric cancer previously treated with a fluoropyrimidine and/or a platinum [30] or a taxane [31], respectively. Despite this modest activity in Western patients, further evaluation of ixabepilone in Asian patients with gastric cancer is warranted based on growing evidence highlighting epidemiological and genetic differences between Asian and Western populations [32]; gene expression profiling revealed differential expression of multiple genes in Eastern versus Western gastric tumor libraries [33]. Moreover, several retrospective analyses have shown that Asian patients are more likely to be diagnosed with localized tumors and have tumors located in the gastric antrum, whereas Western patients are more likely to have distant metastases and a prognostically less favorable tumor location in the cardia [34–36]. In these retrospective cohorts, median survival was longer in Asian patients than in Western patients, likely reflecting the differences in disease characteristics at presentation.

The present phase II study was designed to evaluate the efficacy and safety of single-agent ixabepilone in Asian patients with advanced gastric adenocarcinomas in which prior fluoropyrimidine-based therapy had failed. The primary objective was to determine the ORR; secondary objectives were to assess time to response, duration of response, disease control rate (DCR), PFS, and safety and tolerability.

## Methods

### Patients

Men and women of Asian ethnicity aged ≥18 years with histologically confirmed unresectable or metastatic gastric adenocarcinoma originating in the stomach or gastroesophageal junction were eligible if a fluoropyrimidine-based chemotherapy regimen had failed in an adjuvant, locally advanced, or metastatic setting. Failure of fluoropyrimidine-based chemotherapy was defined by disease progression while receiving such therapy or by disease recurrence within 12 months of the last dose. Eligibility also required measurable disease by response evaluation criteria in solid tumors (RECIST) guidelines (version 1.1) [37], Eastern Cooperative Oncology Group performance status 0–1, adequate hematologic, hepatic, and renal function, and life expectancy >12 weeks. Women of childbearing potential required a negative pregnancy test within 72 h before starting ixabepilone and agreed to use an adequate method of contraception to avoid pregnancy for up to 4 weeks after the last dose. All patients provided written informed consent before participating in this study.

Patients were excluded if they had known central nervous system metastasis or neurological signs and symptoms suggestive of such metastasis, prior taxane or ixabepilone therapy, peripheral neuropathy (≥grade 2), or any significant medical illness precluding systemic anticancer therapy. Patients who had received >1 prior chemotherapy regimen for metastatic disease or >2 prior chemotherapy regimens overall were ineligible. Concurrent anti-cancer treatment including investigational agents was not permitted during this study. Strong CYP3A4 inhibitors (e.g., ketoconazole) were discontinued within 1 week prior to starting study treatment.

### Study design

This phase II, single-arm, open-label study was conducted at 9 sites in Asia including 2 sites in Japan, 3 sites in Korea, 2 sites in Taiwan, and 1 site each in Hong Kong and Singapore from November of 2009 to June of 2011. The study was run in accordance with ethical principles originating in the 1964 Declaration of Helsinki and in compliance with Good Clinical Practice and national regulatory guidelines. The study protocol and informed consent form were approved by the Institutional Review Board or Independent Ethics Committee at each study site before patient enrollment.

Ixabepilone was administered at a dose of 40 mg/m^2^ as a 3-h infusion every 21 days. Premedication with H_1_ and H_2_ antagonists was given to prevent hypersensitivity reactions. Patients who experienced a hypersensitivity reaction were required to receive additional premedication with intravenous corticosteroids before subsequent ixabepilone doses.

Subsequent cycles of ixabepilone were administered after all treatment-related toxicities had resolved to baseline or grade 1 (or ≤grade 2 for alopecia and fatigue), absolute neutrophil counts were ≥1,500 cells/μL, and platelet counts were ≥100,000 cells/μL. Patients who did not meet these criteria were re-evaluated weekly; those who failed to recover within 3 weeks of a scheduled re-treatment were discontinued from protocol treatment. The duration of treatment was based on a tumor assessment done every other cycle starting from the first dose of the study treatment. Patients achieving a complete response (CR) were treated for a maximum of 4 cycles after documentation of CR or up to a maximum of 8 cycles, whichever came first. Patients with stable disease (SD) or a partial response (PR) were treated until disease progression, unacceptable toxicity, or a maximum of 8 cycles.

Patients experiencing certain toxicities had the dose of ixabepilone reduced in subsequent cycles to 32 mg/m^2^, and if toxicity recurred, to 25 mg/m^2^. Toxicities mandating dose reduction were grade 4 neutropenia lasting ≥7 days, febrile neutropenia, grade 4 thrombocytopenia, grade 3 thrombocytopenia with bleeding, grade 2 neuropathy lasting ≥7 days, or grade 3 neuropathy lasting <7 days. The reduced dose was then administered in all subsequent cycles. Ixabepilone was discontinued for toxicity requiring more than 2 dose reductions or in the event of grade 3 neuropathy lasting ≥7 days, disabling neuropathy, or any grade 4 non-hematologic toxicity. Palliative and supportive care for disease-related symptoms was allowed during the study.

### Assessments

Clinical and radiological evaluation (abdominal and chest computed tomography) of treatment response was conducted every other cycle until disease progression was documented. Treatment response was evaluated according to modified RECIST guidelines (version 1.1) [37]. Patients with CRs or PRs were to have repeat tumor assessments within 4–6 weeks to confirm the response. The ORR was the proportion of patients who achieved either a CR or PR; the DCR was the proportion of patients whose best response was CR, PR, or SD. The time to response was defined as the time interval from the first dose of ixabepilone until measurement criteria for PR or CR were first met, whereas the duration of response was defined as the time interval from when measurement criteria for PR or CR were first met until documented progressive disease or death. PFS was defined as the time interval from the first day of treatment until documented progressive disease or death.

A focused physical examination, including neuropathy assessment, was performed within 2 weeks before the first dose of ixabepilone and then prior to each subsequent dose. Serum chemistry and hematology were measured at the same time, whereas blood counts and differentials were ordered weekly during the first 3 cycles and then as clinically indicated to monitor recovery from hematological toxicity. Adverse events were monitored continuously and graded according to the National Cancer Institute Common Terminology Criteria of Adverse Events, version 3.0.

### Statistics

This study used Simon’s 2-stage optimal design to determine whether ixabepilone produces an ORR of clinical interest (>8 %); an ORR ≤8 % was not of clinical interest and an ORR ≥20 % was of strong clinical interest. The first stage required 25 response-evaluable patients. Study termination was planned if ≤2 of the 25 patients responded to treatment; otherwise, an additional 27 response-evaluable patients would be treated. The study required at least 8 responders among the 52 evaluable patients at the end of the second stage to reject the null hypothesis of ORR ≤8 %. The test had 80 % power to reject the null hypothesis at a significance level of 5 % if the true ORR is 20 %.

The ORR and DCR were calculated for all treated patients. For each, a 2-sided 95 % exact confidence interval (CI) was computed using the Clopper–Pearson method. Duration of response and PFS were analyzed by Kaplan–Meier methodology, with computation of median values and their 2-sided 95 % CIs. All other parameters, including time to response, demographic and baseline characteristics, and safety variables, were analyzed with descriptive statistics.

## Results

### Patient disposition and characteristics

Fifty-eight patients were screened, 6 (10.3 %) were not treated because of screening failure, and the remaining 52 patients (89.7 %) were enrolled and received ixabepilone. Of those treated, 4 patients (7.7 %) completed ixabepilone therapy according to the study protocol, 38 patients (73.1 %) discontinued because of disease progression, 5 patients (9.6 %) withdrew consent or requested study drug discontinuation, 4 patients (7.7 %) discontinued because of adverse events, and 1 patient (1.9 %) died.

The median age of the study cohort was 56.5 years (range: 29.0–77.0 years); most were men (65.4 %) and all were of Asian ethnicity (Table 1). The majority of patients had 3 or more disease sites (53.8 %), most frequently in the lymph nodes (71.2 %), stomach (55.8 %), and liver (36.5 %).

**Table 1:** Patient characteristics

Characteristic	*N* = 52
Age, years	
Median (range)	56.5 (29.0–77.0)
≥65 years, *n* (%)	12 (23.1)
Gender, *n* (%)	
Male	34 (65.4)
Female	18 (34.6)
Ethnicity, *n* (%)	
Chinese	23 (44.2)
Japanese	15 (28.9)
Korean	13 (25.0)
Asian other	1 (1.9)
ECOG performance status, *n* (%)	
0	20 (38.5)
1	32 (61.5)
Number of disease sites, *n* (%)	
1	11 (21.2)
2	13 (25.0)
≥3	28 (53.8)
Disease sites, *n* (%)	
Lymph node	37 (71.2)
Gastric	29 (55.8)
Peritoneum (including ascites)	23 (44.2)
Liver	19 (36.5)
Lung	8 (15.4)
Other	30 (57.7)

*ECOG* Eastern Cooperative Oncology Group

### Exposure

Ixabepilone was administered for a median of 3.5 courses (range: 1–10). Of the 45 patients who received at least 2 courses, 18 (40 %) required at least 1 dose reduction of ixabepilone. The reasons for the first dose reduction included hematologic toxicity in 6 patients (13.3 %), neuropathy in 4 patients (8.9 %), and other non-hematologic toxicity in 8 patients (17.8 %).

### Efficacy

**Fig. 1:**
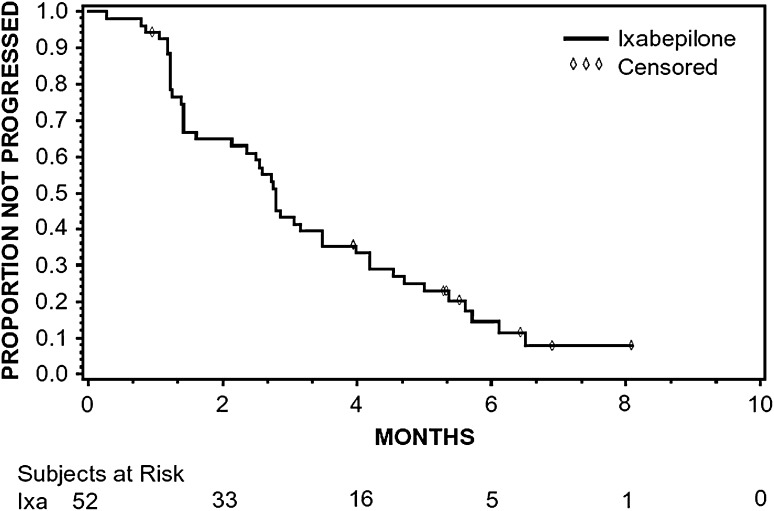
Kaplan–Meier plot of progression-free survival

The ORR with ixabepilone therapy was 15.4 % (95 % CI 6.9–28.1); all objective responses were PR (Table 2). Twenty-six additional patients (50.0 %) had SD and, therefore, the DCR was 65.4 % (95 % CI 50.9–78.0). For patients achieving PR, the median time to response was 8.9 weeks (range: 5.1–12.1 weeks) and the median duration of response was 3.1 months (95 % CI 2.6–4.1 months). Median PFS was 2.8 months (95 % CI 2.1–3.5 months) (Fig. 1).

**Table 2:** Best overall response

Parameter	*N* = 52
Best response, *n* (%)	
CR	0 (0)
PR	8 (15.4)
SD	26 (50.0)
Progressive disease	15 (28.8)
Unable to determine	3 (5.8)
ORR (95 % CI)	15.4 (6.9–28.1)
DCR (95 % CI)	65.4 (50.9–78.0)

### Safety

The adverse events reported were consistent with the known safety profile of ixabepilone. Fifty patients (96.2 %) had at least 1 adverse event, most commonly alopecia, decreased appetite, neutropenia, peripheral sensory neuropathy, and fatigue (Table 3). Most non-hematologic toxicity was grade 1 or 2; the most common grade 3 events were fatigue (9.6 %), decreased appetite (7.7 %), peripheral sensory neuropathy (5.8 %), and diarrhea (5.8 %). Overall, peripheral neuropathies were reported by 33 patients (63.5 %), with the most common forms being peripheral sensory neuropathy (48.1 %) and hypoesthesia (11.5 %). Peripheral motor neuropathy occurred in 1 patient (1.9 %; grade 2). In terms of hematological toxicity, grade 3/4 neutropenia and leukopenia occurred in 24 (46.2 %) and 11 (21.1 %) patients, respectively, with febrile neutropenia in 4 patients (7.7 %). Grade 3 anemia and thrombocytopenia occurred in 3 (5.8 %) and 2 (3.8 %) patients, respectively.

**Table 3:** Treatment-related adverse events (AEs) reported at an incidence ≥10 %

AE	Grade 1	Grade 2	Grade 3	Grade 4	Total
Any AE	7 (13.5)	11 (21.2)	12 (23.1)	19 (36.5)	50 (96.2)^a^
Hematologic AEs					
Neutropenia	0 (0)	2 (3.8)	8 (15.4)	16 (30.8)	26 (50.0)
Leukopenia	0 (0)	1 (1.9)	9 (17.3)	2 (3.8)	12 (23.1)
Non-hematologic AEs					
Alopecia	26 (50.0)	9 (17.3)	0 (0)	0 (0)	35 (67.3)
Decreased appetite	14 (26.9)	11 (21.2)	4 (7.7)	0 (0)	29 (55.8)
Peripheral sensory neuropathy	12 (23.1)	10 (19.2)	3 (5.8)	0 (0)	25 (48.1)
Fatigue	5 (9.6)	12 (23.1)	5 (9.6)	0 (0)	22 (42.3)
Rash	11 (21.2)	5 (9.6)	1 (1.9)	0 (0)	17 (32.7)
Diarrhea	10 (19.2)	1 (1.9)	3 (5.8)	0 (0)	14 (26.9)
Constipation	9 (17.3)	4 (7.7)	0 (0)	0 (0)	13 (25.0)
Nausea	8 (15.4)	4 (7.7)	1 (1.9)	0 (0)	13 (25.0)
Myalgia	9 (17.3)	2 (3.8)	1 (1.9)	0 (0)	12 (23.1)
Arthralgia	7 (13.5)	4 (7.7)	0 (0)	0 (0)	11 (21.2)
Weight decreased	2 (3.8)	9 (17.3)	0 (0)	0 (0)	11 (21.2)
Pruritus	6 (11.5)	3 (5.8)	0 (0)	0 (0)	9 (17.3)
Pyrexia	8 (15.4)	0 (0)	0 (0)	0 (0)	8 (15.4)
Vomiting	5 (9.6)	3 (5.8)	0 (0)	0 (0)	8 (15.4)
Stomatitis	2 (3.8)	3 (5.8)	2 (3.8)	0 (0)	7 (13.5)
Asthenia	1 (1.9)	5 (9.6)	0 (0)	0 (0)	6 (11.5)
Dysgeusia	5 (9.6)	1 (1.9)	0 (0)	0 (0)	6 (11.5)
Hypoesthesia	2 (3.8)	3 (5.8)	1 (1.9)	0 (0)	6 (11.5)
Nail disorder	5 (9.6)	0 (0)	1 (1.9)	0 (0)	6 (11.5)

^a^Includes 1 patient with grade 5 pneumonia and neutropenic sepsis

Four patients (7.7 %) discontinued treatment because of drug-related adverse events, including 3 patients with peripheral neuropathy and 1 patient with febrile neutropenia. There was 1 death because of drug-related toxicity: a 69-year-old male patient died of pneumonia and neutropenic sepsis during course 6 of ixabepilone therapy. The patient started course 6 with a reduced dose of 32 mg/m^2^ because the investigator had considered the patient too weak to continue at the initial dose. The death occurred 18 days after the last treatment. Three other patients died within 30 days of their last dose of ixabepilone, all of which were assessed by the investigator as due to disease progression.

## Discussion

The results of this phase II study demonstrate that ixabepilone has activity of clinical interest when administered at a dose of 40 mg/m^2^ every 21 days to Asian patients with unresectable or metastatic gastric cancer who had progressed on or within 12 months after receiving fluoropyrimidine-based therapy. In this population, ixabepilone produced an ORR of 15.4 % and DCR of 65.4 %. This is in contrast to the lower ORRs of 5 % and 9 % reported for 50 mg/m^2^ ixabepilone administered every 21 days in Western patients with metastatic gastric cancer previously treated with a fluoropyrimidine and/or a platinum [30] or a taxane [31], respectively.

The activity of ixabepilone appears consistent with contemporary studies of taxanes in second-line treatment of Asian patients with advanced gastric cancer. Docetaxel produced ORRs of 14–16 % in phase II trials conducted in Korea [15, 38]. In the largest of these studies, docetaxel was administered to 154 patients who had failed fluoropyrimidine and platinum therapy, of whom 86 were evaluable for response; the ORR and DCR were 14 and 43 %, respectively, and median time to progression was 2.6 months [38]. Rates up to 24 % were reported for docetaxel in Japanese patients with recurrent or metastatic gastric cancer, but these studies were conducted more than a decade ago and, consequently, patients may not have received optimal initial chemotherapy [39, 40]. In a recent Japanese study, biweekly paclitaxel after failure of fluoropyrimidine-based therapy produced an ORR of 17.5 % and DCR of 70.0 % with a median PFS of 3.6 months [16]. Besides taxanes, other cytotoxic agents including irinotecan have shown similar activity in advanced gastric cancer [41], whereas various targeted agents have shown modest single-agent activity in this setting [42].

Although multiple drugs have been evaluated as second-line therapy in phase II trials and retrospective cohorts, there have been no randomized head-to-head trials designed to establish a standard treatment in this setting [43]. Comparisons of second-line therapy across clinical studies are problematic for multiple reasons, including the nature of previous chemotherapy and responses to first-line chemotherapy [13]. This is particularly important in advanced gastric cancer since response duration to first-line chemotherapy is prognostic for the benefit of second-line chemotherapy [44, 45]. With targeted agents being increasingly tested in conjunction with first-line chemotherapy, it will be important to evaluate how they impact the activity of subsequent second-line treatment and, conversely, how second-line therapy affects outcomes measured with first-line regimens [43].

Current treatment options in second-line advanced gastric cancer provide only small overall survival (OS) benefit over best supportive care (BSC). A recent randomized phase III trial of 193 Asian patients assessed the efficacy and safety of BSC combined with either docetaxel (60 mg/m^2^ every 3 weeks) or irinotecan (150 mg/m^2^ every 2 weeks) compared with BSC alone as a second-line therapy in advanced gastric cancer [17]. The OS of patients randomized to BSC plus docetaxel or irinotecan (*n* = 128) versus BSC alone (*n* = 65) was 5.1 and 3.8 months, respectively; the difference was statistically significant (hazard ratio, 0.63; 95 % CI 0.47–0.86; *P* = 0.004) and was maintained in most of the prospectively defined subgroups including age, gender, performance status, number of prior treatments, number of metastatic sites, hemoglobin levels, and response to prior chemotherapy. Docetaxel or irinotecan improves OS when added to BSC in second-line advanced gastric cancer, but the OS improvement of 1.3 months over BSC only underscores the current unmet medical need for more efficient treatments in this patient population. Another recent phase III trial comparing single-agent irinotecan versus BSC in Germany was closed prematurely after accrual of only 40 patients [18]. Irinotecan produced no objective responses and SD in 53 %, but showed a statistically significant improvement in median OS (4.0 vs 2.4 months; *P* = 0.012).

In Asian gastric cancer patients, ixabepilone showed a safety profile similar to that previously reported in other tumor types. Grade 3/4 toxicity consisted mostly of neutropenia, whereas the most clinically relevant treatment-related non-hematological adverse events were decreased appetite (anorexia), peripheral sensory neuropathy, and fatigue, mostly grade 1 or 2 in severity. In an earlier study conducted in Western patients with gastric cancer, nausea, fatigue, sensory neuropathy, vomiting, and anorexia were commonly seen with ixabepilone given every 3 weeks at a higher dosage (50 mg/m^2^) than the one used in this study; frequencies of each of these events except for fatigue reduced when a lower ixabepilone dose was administered over a 5-day period every 3 weeks [31]. At the dose used in this study (40 mg/m^2^ every 3 weeks, the approved regimen in breast cancer), the incidence of peripheral sensory neuropathy and fatigue was consistent with rates seen in clinical trials of other tumor types and in other studies of recurrent disease, including breast cancer [19, 20] and endometrial carcinoma [27]. Gastrointestinal adverse events were also common across tumor types, although the nature of these events (e.g., anorexia, nausea) varied in incidence. In general, the safety profile of ixabepilone is better in earlier lines of therapy as demonstrated in the TITAN study of patients with metastatic breast cancer treated in a first-line setting [46].

In summary, ixabepilone showed clinical activity with an ORR of 15.4 % in Asian patients with unresectable or metastatic gastric cancer in whom fluoropyrimidine-based chemotherapy had failed. Ixabepilone therapy was tolerable for most patients and its safety profile was similar to that previously reported in other tumor types.
